# Functional mechanisms of MYRF DNA-binding domain mutations implicated in birth defects

**DOI:** 10.1016/j.jbc.2021.100612

**Published:** 2021-03-30

**Authors:** Chuandong Fan, Hongjoo An, Mohamed Sharif, Dongkyeong Kim, Yungki Park

**Affiliations:** Hunter James Kelly Research Institute, Department of Biochemistry, Jacobs School of Medicine and Biomedical Sciences, State University of New York at Buffalo, Buffalo, New York, USA

**Keywords:** MYRF, membrane-bound transcription factor, transcription factor, mutation, birth defects, disease mechanism, DBD, DNA-binding domain, ICA, Intramolecular Chaperone Auto-processing, Myrf, myelin regulatory factor, OL, oligodendrocyte, OPC, OL progenitor cell

## Abstract

Myrf is a pleiotropic membrane-bound transcription factor that plays critical roles in diverse organisms, including in oligodendrocyte differentiation, embryonic development, molting, and synaptic plasticity. Upon autolytic cleavage, the Myrf N-terminal fragment enters the nucleus as a homo-trimer and functions as a transcription factor. Homo-trimerization is essential for this function because it imparts DNA-binding specificity and affinity. Recent exome sequencing studies have implicated four *de novo* MYRF DNA-binding domain (DBD) mutations (F387S, Q403H, G435R, and L479V) in novel syndromic birth defects involving the diaphragm, heart, and the urogenital tract. It remains unknown whether and how these four mutations alter the transcription factor function of MYRF. Here, we studied them by introducing homologous mutations to the mouse Myrf protein. We found that the four DBD mutations abolish the transcriptional activity of the Myrf N-terminal fragment by interfering with its homo-trimerization ability by perturbing the DBD structure. Since the Myrf N-terminal fragment strictly functions as a homo-trimer, any loss-of-function mutation has the potential to act as a dominant negative. We observed that one copy of Myrf-F387S, Myrf-Q403H, or Myrf-L479V, but not Myrf-G435R, was tolerated by the Myrf N-terminal homo-trimer for structural and functional integrity. These data suggest that F387S, Q403H, and L479V cause birth defects by haploinsufficiency, while G435R does so *via* dominant negative functionality.

Myrf (myelin regulatory factor, previously known as C11orf9 in human and Mrf or Gm98 in mouse) is a membrane-bound transcription factor that plays an important role in diverse organisms ranging from human to slime mold (1, 2, 3, 4, 5, 6, 7). Throughout this paper, for the sake of clarity, *MYRF* and *Myrf* refer to the human and mouse genes, respectively. Likewise, MYRF and Myrf refer to the human and mouse proteins, respectively. In the central nervous system (CNS), the expression of *Myrf* is restricted to oligodendrocytes (OLs), which generate myelin sheaths (1, 8). Conditional knockout of *Myrf* in OL lineage cells blocks the differentiation of OL progenitor cells (OPCs) into OLs, resulting in lethal dysmyelination (1). Myrf is also required for the life-long maintenance, plasticity, and regeneration of myelin in the CNS (9, 10, 11). These studies left the impression that Myrf is a “myelin” transcription factor, and hence the name Myrf. It is now clear that *MYRF* is also expressed in other tissues such as developing diaphragm, heart, stomach, pancreas, and eye (12, 13, 14, 15, 16). Consistently, *MYRF* mutations have been implicated in both myelin and nonmyelin diseases (13, 14, 15, 17, 18, 19, 20), and whole-body *Myrf* knockout leads to embryonic lethality independent of OL generation (1). In keeping with extra-myelin functions of Myrf, Myrf orthologs are found in organisms without myelin, where they are required for cell differentiation, molt, and synaptic plasticity (2, 3, 6, 7).

Myrf is generated as a type-II membrane protein in the endoplasmic reticulum (ER) (4, 5, 6). The Intramolecular Chaperone Auto-processing (ICA) domain induces the homo-trimerization of Myrf (4) (Fig. 1). Powered by the ICA homo-trimer, homo-trimeric Myrf undergoes auto-cleavage, releasing its N-terminal fragment from the ER membrane as a homo-trimer (4, 5, 21, 22). Myrf N-terminal homo-trimer enters the nucleus to work as a transcription factor (Fig. 1) (22, 23, 24, 25). Our previous study showed that Myrf N-terminal fragment strictly works as a homo-trimer (22). Mutations that disrupt the homo-trimerization of Myrf N-terminal fragment abolish its transcriptional activity (22), and this is how Myrf-G575R, a deleterious mutation isolated by a chemical mutagenesis screen in *C. elegans* (2), works.

**Figure 1:**
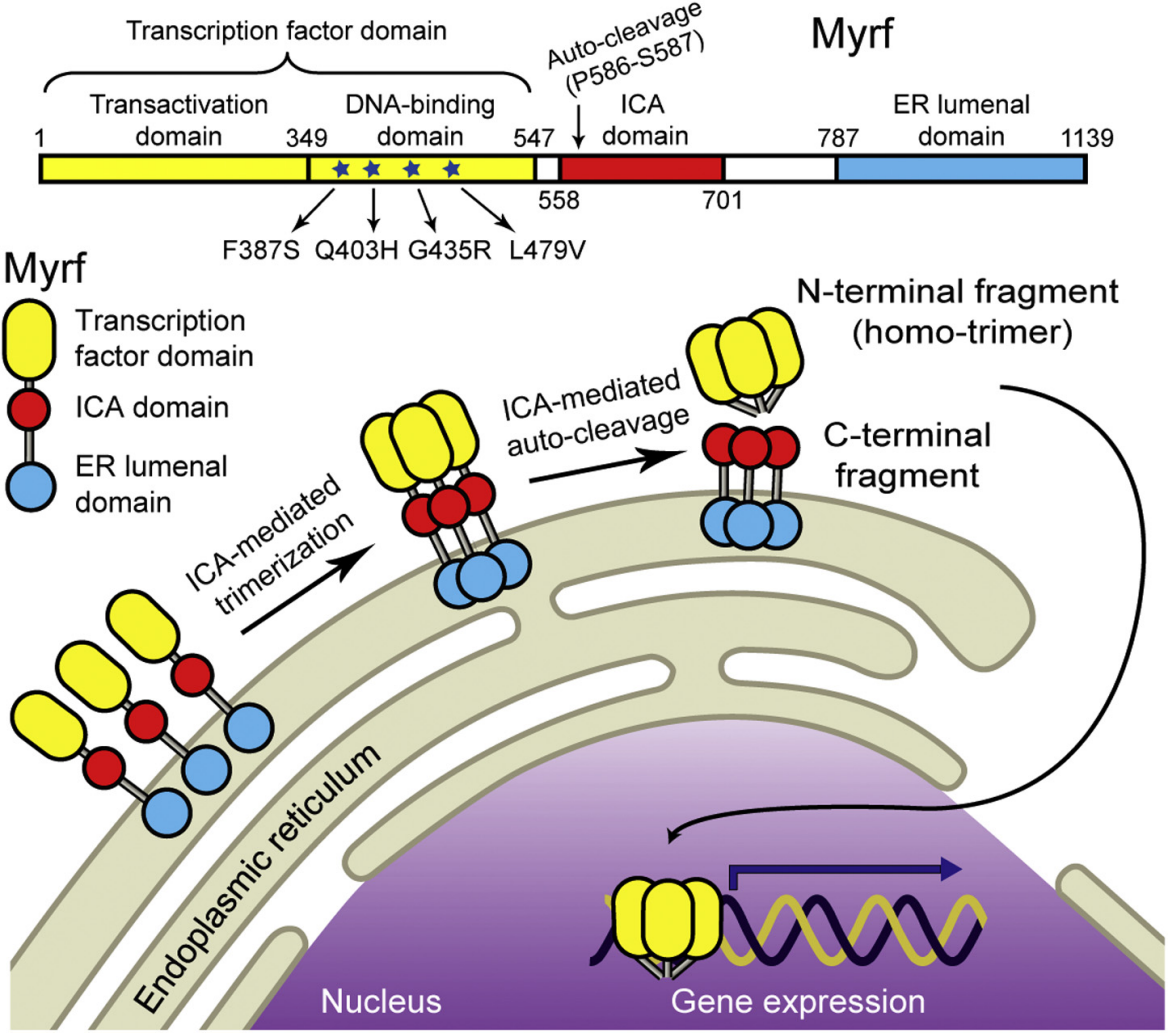
**(*Top*) The domain organization of Myrf and the location of the four MYRF DBD mutations.** (*Bottom*) The schematic of Myrf auto-cleavage.

Several *de novo MYRF* mutations have been identified for congenital anomalies (13, 14, 15, 17, 18, 19, 20). These commonly involve defects in the heart, lung, and urogenital tract and are thought to constitute a novel syndrome. Of the six *MYRF* missense mutations implicated in congenital anomalies, two (V679A and R695H) and four (F387S, Q403H, G435R, and L479V) are mapped to the ICA and DNA-binding domains (DBDs), respectively (Fig. 1). We have recently elucidated the functional mechanisms of the two ICA domain mutations (26). Little is known about whether and how the four DBD mutations affect the transcription factor function of MYRF. If they turn out to be loss-of-function mutations, another issue to address is how they cause birth defects in a heterozygous state. *De novo* mutations are heterozygous by definition, and thus the four DBD mutations are believed to cause birth defects in a heterozygous state. There are two possibilities for a deleterious *de novo* mutation to cause disease—haploinsufficiency or dominant negativity. It remains unknown whether the four MYRF DBD mutations act by haploinsufficiency or dominant negativity. This paper addresses these key issues, revealing that the four DBD mutations abolish the transcriptional activity of Myrf N-terminal fragment by disrupting its homo-trimerization. Our data also suggest that F387S, Q403H, and L479V cause congenital anomalies by haploinsufficiency while dominant negativity is at work for G435R-linked birth defects.

## Results

### Effect of F387S, Q403H, G435R, and L479V on the auto-cleavage of Myrf

**Figure 2:**
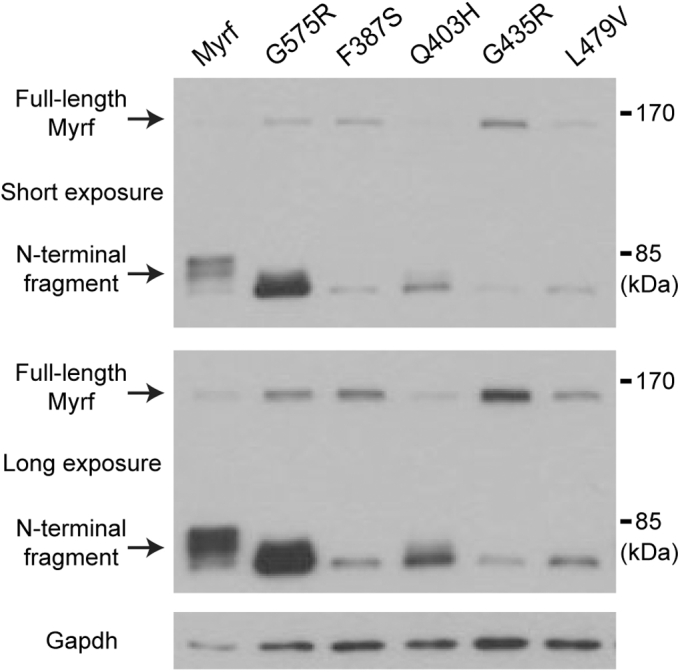
**Western blot of wild-type Myrf, Myrf-G575R, and the four DBD mutants.** These were expressed in Oli-neu cells, and whole-cell lysates analyzed by western blot. Gapdh was used as a loading control.

**Figure 3:**
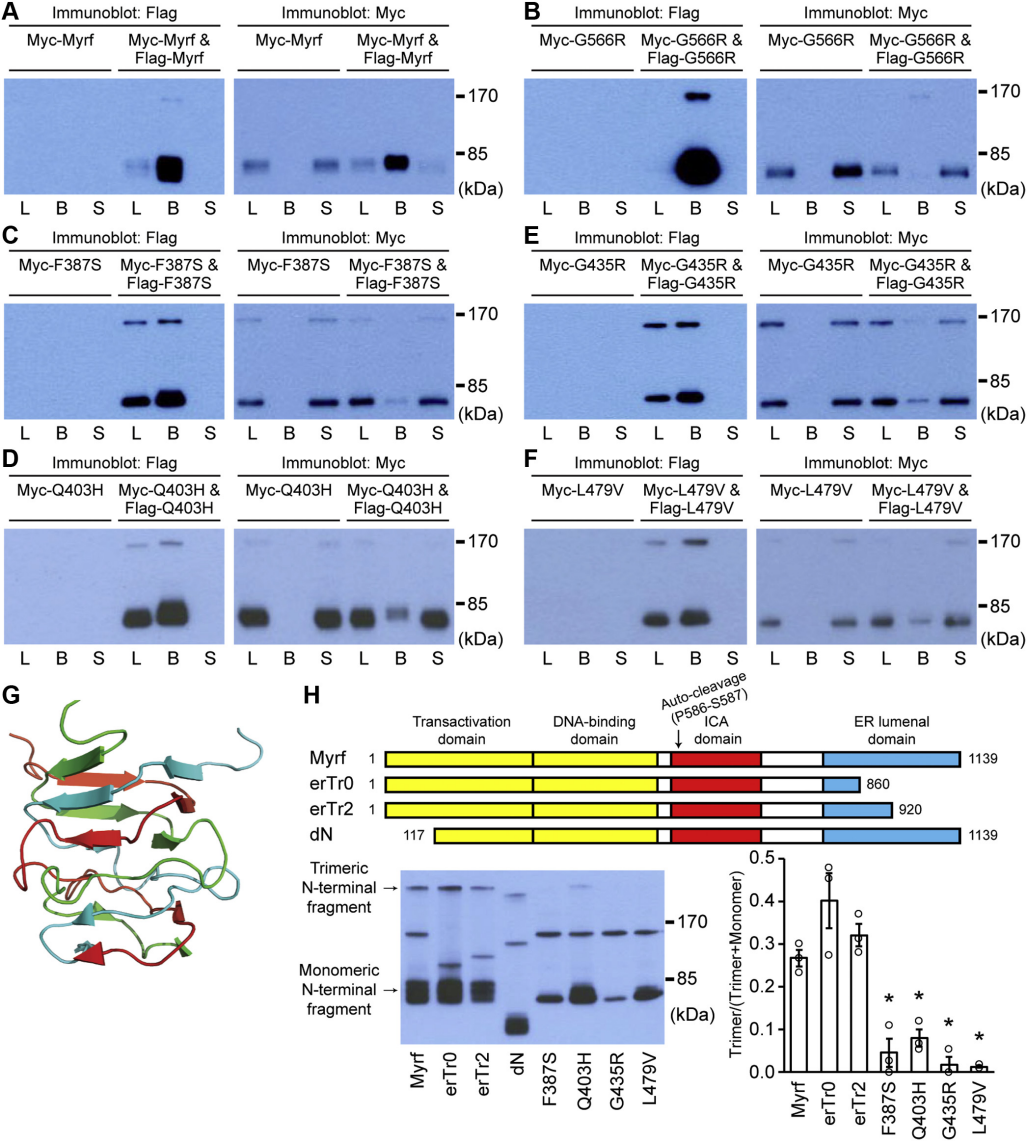
**Co-immunoprecipitation of wild-type Myrf (*A*), MYRF-G566R (*B*), and the four DBD mutants (*C–F*).** These were expressed in HEK293FT cells, and whole-cell lysates analyzed by immunoprecipitation. L: cell lysate used for immunoprecipitation. B: bead fraction after immunoprecipitation. S: supernatant fraction after immunoprecipitation. *G*, triple β-helix (PDB ID: 3GW6). Image was rendered by PyMOL (The PyMOL Molecular Graphics System Version 1.7 Schrödinger LLC.). *H*, control Myrf constructs used for cold western blot (*top*). Cold western blot result (*bottom left*). The Myrf constructs were tagged with Flag tag at the N terminus and expressed in Oli-neu cells. Upon SDS-PAGE in a cold condition, the membrane was probed with Flag antibodies. Quantification of the cold western blot results (*bottom right*). Shown are data points and their mean and standard error. ∗*p* < 2.73 × 10^-2^ by two-sided Student’s *t*-test corrected by the Bonferroni procedure (comparison to Myrf).

**Figure 4:**
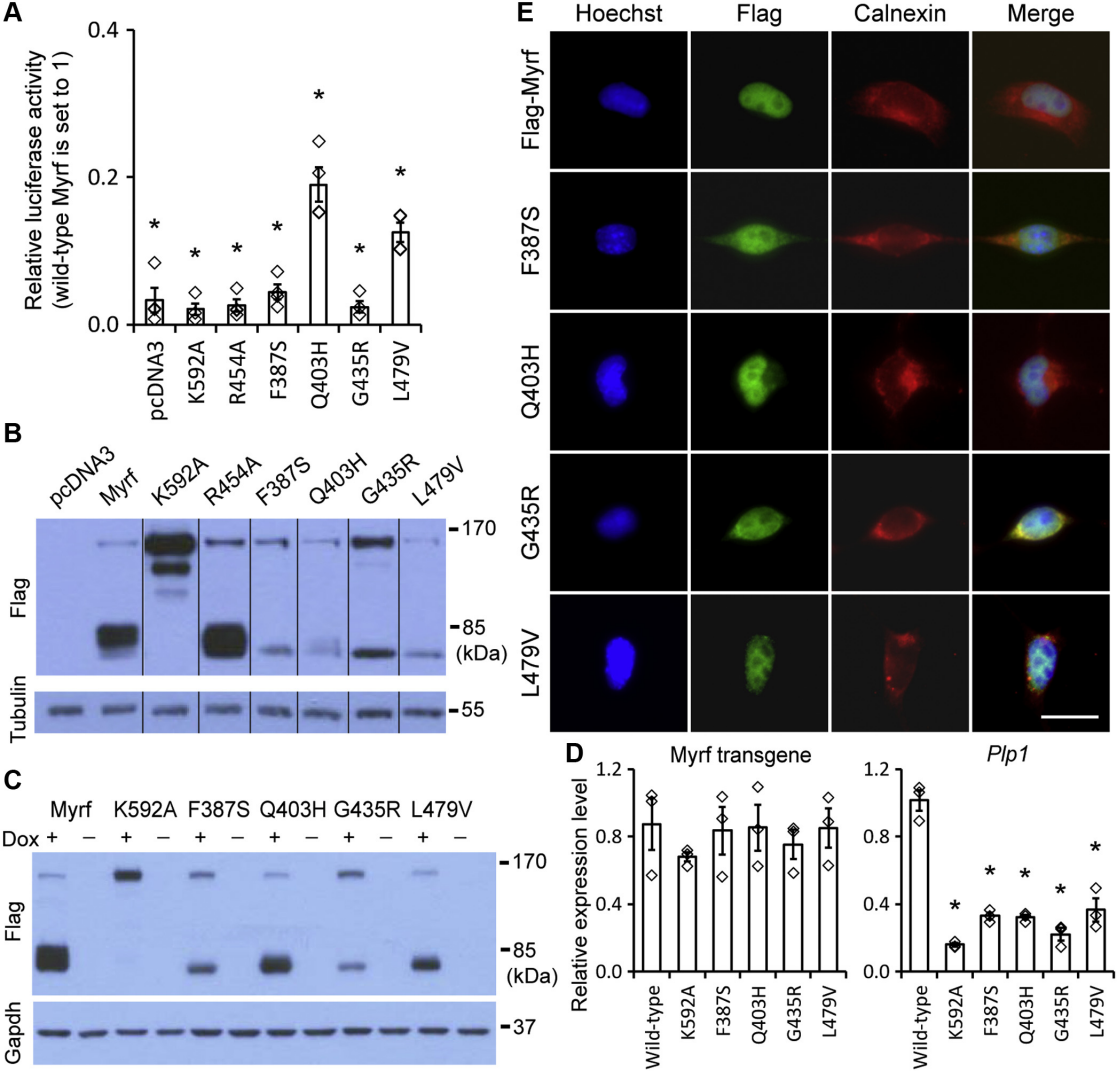
**Luciferase assay, gene expression analysis, and immunofluorescence of wild-type Myrf and the four DBD mutants.***A*, the transcriptional activity of each construct was compared with that of wild-type Myrf by luciferase assay. Rffl was used as a reporter, and the reporter activity for wild-type Myrf was set to 1. Shown are data points and their mean and standard error. ∗*p* < 3.70 × 10^-4^ by two-sided one sample Student’s *t*-test with Bonferroni correction. *B*, Western blot of the luciferase assay sample. The grouping of cropped blots is indicated by the dividing lines. The raw Western blot results are available in Fig. S1, where cropped portions are marked by yellow boxes. *C*, each construct, N-terminally tagged with Flag tag, was integrated into the genome of Oli-neu cells. Their dox-dependent expression was confirmed by Western blot. *D*, RT-qPCR analysis of Myrf transgenes and *Plp1*. Shown are data points and their mean and standard error. ∗*p* < 1.04 × 10^-2^ by two-sided Student’s *t*-test corrected by the Bonferroni procedure (comparison to the wild-type). *E*, Flag-Myrf, Flag-Myrf-F387S, Flag-Myrf-Q403H, Flag-Myrf-G435R, and Flag-Myrf-L479V were expressed in Oli-neu cells, and the subcellular localization of their N termini determined by immunofluorescence with Flag antibodies. Calnexin is an ER marker. Scale bar, 10 μm.

Exome sequencing studies have implicated four MYRF DBD mutations in congenital anomalies: F387S, Q403H, L479V, and G435R. These four mutations are not found in nominally healthy individuals according to the NHLBI GO Exome Sequencing Project, the 1000 Genomes Project (27), and the ExAC database (28). Thus, they have been assumed, but not proved, to be deleterious mutations. For Myrf to function as a transcription factor, it must undergo auto-cleavage to release its N-terminal fragment from the ER membrane as a homo-trimer (4, 5, 22). Thus, we first checked whether the four mutations impact the auto-cleavage of Myrf. Oli-neu cells, a widely used OL cell line (29), were transfected with Flag-Myrf-F387S (Myrf-F387S with an N-terminal Flag tag), Flag-Myrf-Q403H, Flag-Myrf-G435R, and Flag-Myrf-L479V. Flag-Myrf (wild-type Myrf) was used as a control. Whole-cell lysates were subject to immunoblotting with Flag antibodies. Flag-Myrf underwent auto-cleavage efficiently such that full-length Myrf was almost invisible, and three distinct species were resolved for its N-terminal fragment (Fig. 2). The four Myrf mutants were also proteolytically processed, indicating that the four mutations do not block the auto-cleavage of Myrf. Strikingly, however, their N-terminal fragments were all resolved as a single species (Fig. 2), unlike wild-type Myrf N-terminal fragment. Our previous study showed that Myrf N-terminal fragment undergoes post-translational modifications in a manner that depends on its homo-trimerization (22). This is why three distinct species are observed for wild-type Myrf N-terminal fragment while monomeric Myrf N-terminal fragment (*e.g.*, that of Myrf-G575R in Fig. 2) is resolved as a single species. The resolving pattern of mutant Myrf N-terminal fragments strongly suggests that they exist as monomers. If so, it would explain their pathogenic mechanism because homo-trimerization is essential for the transcriptional activity of Myrf N-terminal fragment. Of note, the auto-cleavage efficiency of Myrf-G435R appears to be lower than that of the other three mutants, and this was repeatedly observed throughout our study (*e.g.*, Figs. 3*H* and 4*C*). Thus, in addition to impairing the homo-trimerization of Myrf N-terminal fragment, the G435R mutation also seems to interfere with the auto-cleavage reaction in the ER membrane.

### Effect of F387S, Q403H, G435R, and L479V on the homo-trimerization of Myrf N-terminal fragment

The immunoblotting results predict that the N-terminal fragments of Myrf-F387S, Myrf-Q403H, Myrf-G435R, and Myrf-L479V fail to form homo-trimers. To test this hypothesis, we performed co-immunoprecipitation experiments. To demonstrate the effectiveness and specificity of co-immunoprecipitation, we first tested the homo-oligomerization of wild-type Myrf and MYRF-G566R (equivalent to *C. elegans* Myrf-G575R). Myc-Myrf (wild-type Myrf with an N-terminal Myc tag) was transfected into Oli-neu cells, either alone or together with Flag-Myrf. Cell lysates (“L” in Fig. 3*A*) were subject to immunoprecipitation with Flag beads, resulting in the bead and sup fractions (“B” and “S” in Fig. 3*A*). The N-terminal fragment of Myc-Myrf did not bind to Flag beads (Fig. 3*A*). When coexpressed with Flag-Myrf, however, it avidly bound to Flag beads, consistent with its homo-trimerization. In contrast, the N-terminal fragment of Myc-MYRF-G566R did not bind to Flag beads even in the presence of Flag-MYRF-G566R (Fig. 3*B*), in line with the previous observation that MYRF-G566R N-terminal fragment exists as a monomer (22). These control experiments set the stage for the test of the four mutants. The N-terminal fragment of Myc-Myrf-F387S was mostly detected in the sup fraction, even when coexpressed with Flag-Myrf-F387S (Fig. 3*C*), indicating that a majority of Myrf-F387S N-terminal fragments exist as a monomer. The same was also true for the other three mutants (Fig. 3, *D*–*F*). These results indicate that, even under the overexpression condition, the N-terminal fragments of Myrf-F387S, Myrf-Q403H, Myrf-G435R, and Myrf-L479V hardly form homo-trimers.

In an effort to quantify the effect of the four mutations on the trimerization of Myrf N-terminal fragment, we performed cold Western blot. It takes advantage of the fact that homo-trimerization of Myrf N-terminal fragment is driven by triple-β helix (22). Triple β-helix is a structural motif where three polypeptide chains are intertwined around a common threefold axis (Fig. 3*G*), leading to strong trimerization. Thanks to the triple-β helix, the homo-trimer of Myrf N-terminal fragment survives SDS-PAGE in a cold chamber, as shown by our previous study (22). Upon cold SDS-PAGE, one can measure the signal intensity of trimeric versus monomeric Myrf N-terminal fragments (Fig. 3*H*) and quantify the impact of mutations. Further, since all samples are analyzed simultaneously under the same condition, an objective comparison of wild-type and mutant Myrf species is feasible. We used two variants (erTr0 and erTr2, Fig. 3*H*) as controls to validate our experimental scheme. Since they generate the same N-terminal fragment as wild-type Myrf, the ratio between trimeric and monomeric N-terminal fragments should be comparable for wild-type Myrf, erTr0, and erTr2. Indeed, no significant difference in the ratio was observed for the three species (Fig. 3*H*). In contrast, the ratio was much lower for the four mutants compared with wild-type Myrf (Fig. 3*H*, ∗*p* < 2.73 × 10^-2^ by Student’s *t*-test corrected by the Bonferroni procedure). These results reinforce our conclusion from the co-immunoprecipitation experiments that the four mutations significantly disrupt the homo-trimerization of Myrf N-terminal fragment.

### Effect of F387S, Q403H, G435R, and L479V on the transcriptional activity of Myrf

Since homo-trimerization is essential for the transcriptional activity of Myrf N-terminal fragment, the preceding results predict that the four mutations would abolish the transcriptional activity of Myrf. To test this hypothesis, we performed a luciferase assay in Oli-neu cells with Rffl. Rffl is a highly specific and sensitive Myrf luciferase reporter (5, 22, 25), which was generated by cloning an Myrf ChIP-seq peak in the *Rffl* locus (rn4 chr10:71034166–71034749) into pGL3 promoter. In this assay, we transfected only 2 ng of DNA plasmids for 24-well plates to minimize overexpression artifacts. The reporter activity for Flag-Myrf was set to 1. As expected, the reporter activity for pcDNA3 (an empty vector control), Flag-Myrf-K592A (a mutant Myrf that does not undergo auto-cleavage (4, 5)), and Flag-Myrf-R454A (a mutant Myrf that does not bind to DNA (5)) was much lower (Fig. 4*A*). Flag-Myrf-F387S, Flag-Myrf-Q403H, Flag-Myrf-G435R, and Flag-Myrf-L479V failed to elevate the reporter activity of Rffl (Fig. 4*A*). Western blot analysis of the luciferase assay samples showed that the mutant Myrf constructs were expressed well (Fig. 4*B*), ruling out the trivial possibility that the blunted transcriptional activity of the mutant Myrf species was due to poor protein expression.

We wanted to complement the luciferase assay with a gene expression analysis to show that the expression of endogenous Myrf target genes is affected by the four DBD mutations. To this end, we generated Oli-neu cell lines that express wild-type and mutant Myrf species in a doxycycline-dependent manner. Constitutive expression of Myrf is not compatible with the proliferation of Oli-neu cells, and thus it was necessary to generate inducible cell lines. We included Myrf-K592A as a negative control. By using the Sleeping Beauty transposon system (30), Myrf transgenes were integrated into the genome at a low copy number to mimic physiological conditions as much as possible. Altogether, six inducible cell lines were generated (wild-type Myrf, K592A, and the four DBD mutants), and their dox-dependent expression of Myrf transgenes was confirmed by Western blot (Fig. 4*C*). To minimize the potential interference of endogenous *Myrf*, the expression of Myrf transgenes was induced for 1 day in the proliferation condition. RT-qPCR showed that the six Myrf transgenes were induced at comparable levels (Fig. 4*D*). Another project in the lab has found that *Plp1* is a highly sensitive Myrf target gene (manuscript in preparation). Thus, we checked the expression level of *Plp1* in these cell lines. Our RT-qPCR analysis revealed that *Plp1* was expressed at a significantly lower level in the five mutant cell lines (K592A and the four DBD mutants) than in the wild-type cell line, confirming our conclusion from the luciferase assay that the four mutations cripple the transcriptional activity of Myrf.

We and others have shown that Myrf N-terminal fragment, once released from the ER membrane, enters the nucleus regardless of its oligomerization status (4, 5, 22). This nuclear entry is directed by two nuclear localization signals (K_254_KRK and K_491_KGK). Since none of the four DBD mutations (F387S, Q403H, G435R, and L479V) affects the two nuclear localization signals, their N-terminal fragments are expected to be localized to the nucleus. To confirm this, we performed immunofluorescence experiments. Oli-neu cells were transfected with Flag-Myrf-F387S, Flag-Myrf-Q403H, Flag-Myrf-G435R, and Flag-Myrf-L479V, and the subcellular localization of their N termini determined by immunocytochemistry with Flag antibodies. Hoechst and calnexin were used as the nuclear and ER markers, respectively. The N terminus of Flag-Myrf was found almost exclusively in the nucleus (Fig. 4*E*), as reported before (4, 5). The N termini of the four mutants were also detected in the nucleus (Fig. 4*E*), arguing against the possibility that the poor transcriptional activity of the four Myrf mutants is due to nuclear exclusion. Taken together, we conclude that F387S, Q403H, G435R, and L479V abrogate the transcriptional activity of Myrf N-terminal fragment by disrupting its homo-trimerization.

### Molecular basis for the mutational effects of F387S, G435R, L479V, and Q403H

**Figure 5:**
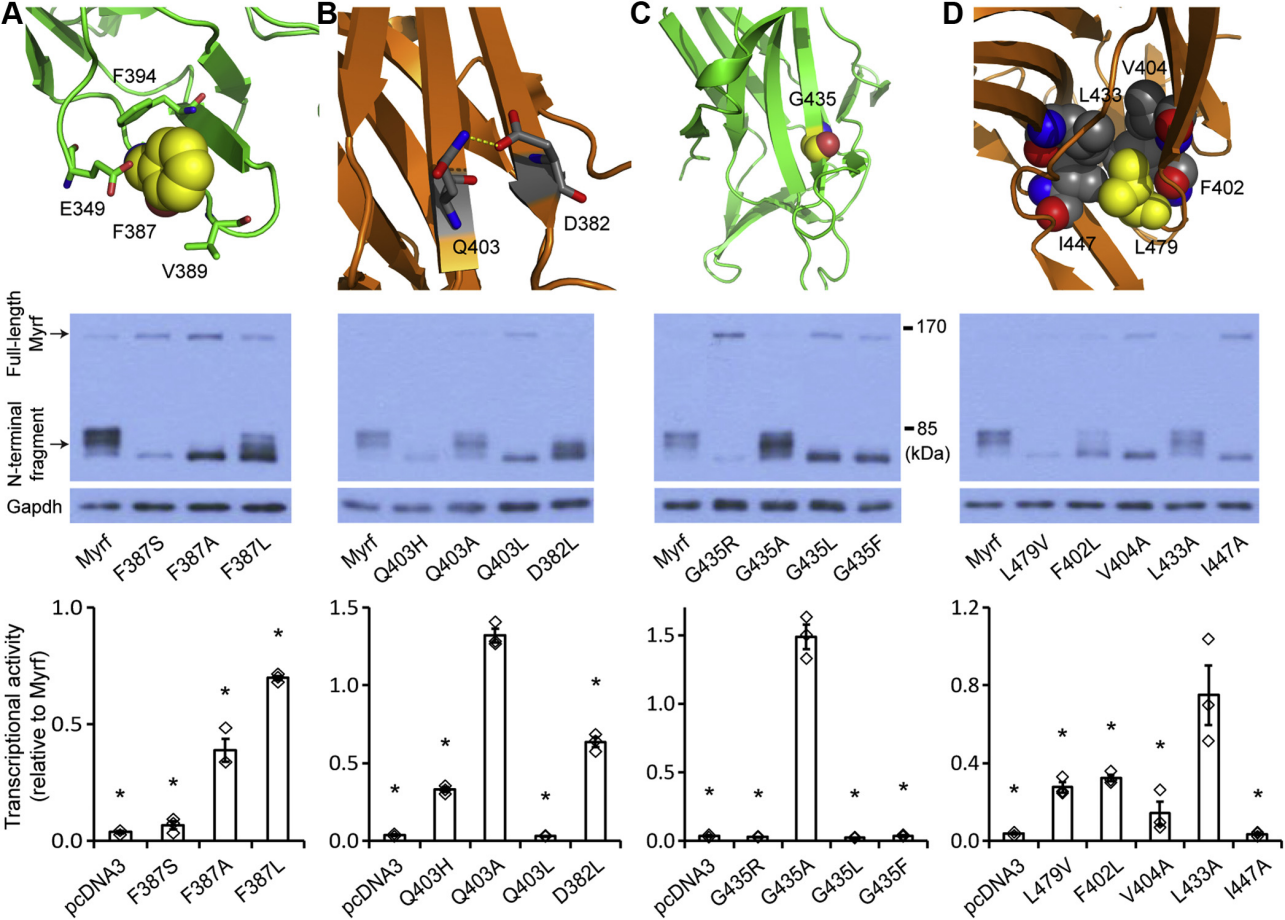
**Molecular mechanisms of the four DBD mutations.***A*, the location of F387 in the Myrf DBD structure. Western blot and luciferase assay of the related Myrf constructs (Myrf-F387A and Myrf-F387L). *B*, the location of Q403 in the Myrf DBD structure. Western blot and luciferase assay of the related Myrf constructs (Myrf-Q403A, Myrf-Q403L, and Myrf-D382L). *C*, the location of G435 in the Myrf DBD structure. Western blot and luciferase assay of the related Myrf constructs (Myrf-G435A, Myrf-G435L, and Myrf-G435F). *D*, the location of L479 in the Myrf DBD structure. Western blot and luciferase assay of the related Myrf constructs (Myrf-F402L, Myrf-V404A, Myrf-L433A, and Myrf-I447A). The PDB ID of the Myrf DBD structure is 5H5P. Images were rendered by PyMOL. For all luciferase assay results, data points and their mean and standard error are shown. ∗*p* < 3.70 × 10^-2^ by two-sided one sample Student’s *t*-test with Bonferroni correction.

To gain insight into the mechanistic mechanisms of the four DBD mutations, we analyzed an Myrf DBD X-ray crystal structure (PDB ID: 5H5P (23)). The crystal structure shows that the side chain of F387 makes a packing interaction with other hydrophobic moieties such as the side chains of V389 and F394 and the aliphatic group of E349 side chain (Fig. 5*A*). Replacement of F387 by serine would perturb this packing interaction. To test this hypothesis, we examined two rationally designed mutants (F387A and F387L). F387A is expected to be detrimental because the side chain of alanine is much smaller than that of phenylalanine, leaving void in the packed space. Yet it is expected to be better than F387S because the side chain of serine is polar, whereas that of alanine is not. F387L would be much more benign than F387S and F387A because the side chain of leucine is bulkier and hydrophobic. Our Western blot showed that Myrf-F387A N-terminal fragment is resolved as a single species (Fig. 5*A*), mirroring the pattern observed for Myrf-F387S N-terminal fragment. Myrf-F387L N-terminal fragment exhibited additional species with a slower electrophoretic mobility (Fig. 5*A*), suggesting a better structural integrity. However, it does not fully match the pattern observed for wild-type Myrf N-terminal fragment, indicating that leucine cannot replace F387 for the integrity of the Myrf DBD structure. To corroborate these mechanistic inferences, we performed a luciferase assay with Rffl as above. The transcriptional activity of Myrf-F387A was about 39% of that of wild-type Myrf (Fig. 5*A*), which is much higher than that of Myrf-F387S. Consistent with the Western blot result, Myrf-F387L exhibited an even higher transcriptional activity, reaching 70% of the wild-type value.

The crystal structure suggests that Q403 makes a hydrogen bond with D382; the distance between the nitrogen atom of Q403 side chain and the oxygen atom of D382 side chain is 2.8 Å (dotted yellow line in Fig. 5*B*). This may explain why Q403H is deleterious. To test this hypothesis, we examined three rationally designed mutants (Q403A, Q403L, and D382L). They are all expected to be detrimental because they would abolish the putative hydrogen bond between Q403 and D382. The electrophoretic mobility of Myrf-Q403L and Myrf-D382L deviated from that of wild-type Myrf (Fig. 5*B*), suggesting that Q403L and D382L, like Q403H, destabilize the Myrf DBD structure. Unexpectedly, Myrf-Q403A N-terminal fragment exhibited an electrophoretic mobility that resembles the wild-type pattern (Fig. 5*B*), suggesting that the Myrf DBD structure somehow tolerates alanine better than histidine and leucine for Q403. Significant structural rearrangement may be induced upon the replacement of Q403 with alanine. In line with these Western blot results, Myrf-Q403A was as capable of activating the reporter activity of Rffl as wild-type Myrf (Fig. 5*B*).

G435 is found between two β strands that form a β sheet. Its replacement by arginine would disrupt the β sheet and block the proper folding of the Myrf DBD. To test this hypothesis, we examined three rationally designed mutants (G435A, G435L, and G435F), where G435 is substituted by an increasingly bulkier hydrophobic moiety. Our Western blot showed that G435L and G435F are as detrimental to the Myrf DBD structure as G435R (Fig. 5*C*). Alanine seems tolerable in light of a wild-type-like electrophoretic mobility for Myrf-G435A N-terminal fragment. Congruently, the luciferase assay showed that Myrf-G435A is as good as wild-type Myrf in elevating the reporter activity of Rffl (Fig. 5*C*). In contrast, the transcriptional activity of Myrf-G435L and Myrf-G435F was as low as that of Myrf-G435R and pcDNA3.

The core structure of the Myrf DBD is two β sheets that pack against each other. L479 is buried between the two β sheets (Fig. 5*D*), likely serving as a hydrophobic glue for them. The crystal structure suggests that L479 interacts with other hydrophobic residues to this end, such as F402, V404, L433, and I447. To test this hypothesis, we examined four rationally designed mutants (F402L, V404A, L433A, and I447A), where each hydrophobic moiety is replaced by a smaller one to compromise the packing interaction. Our Western blot and luciferase assay showed that these mutations except for L433A are not tolerated by the Myrf DBD structure (Fig. 5*D*), highlighting the importance of the L479-centered packing interaction for the structural and functional integrity of the Myrf DBD.

### Structural impact of including Myrf mutants in Myrf N-terminal homo-trimer

**Figure 6:**
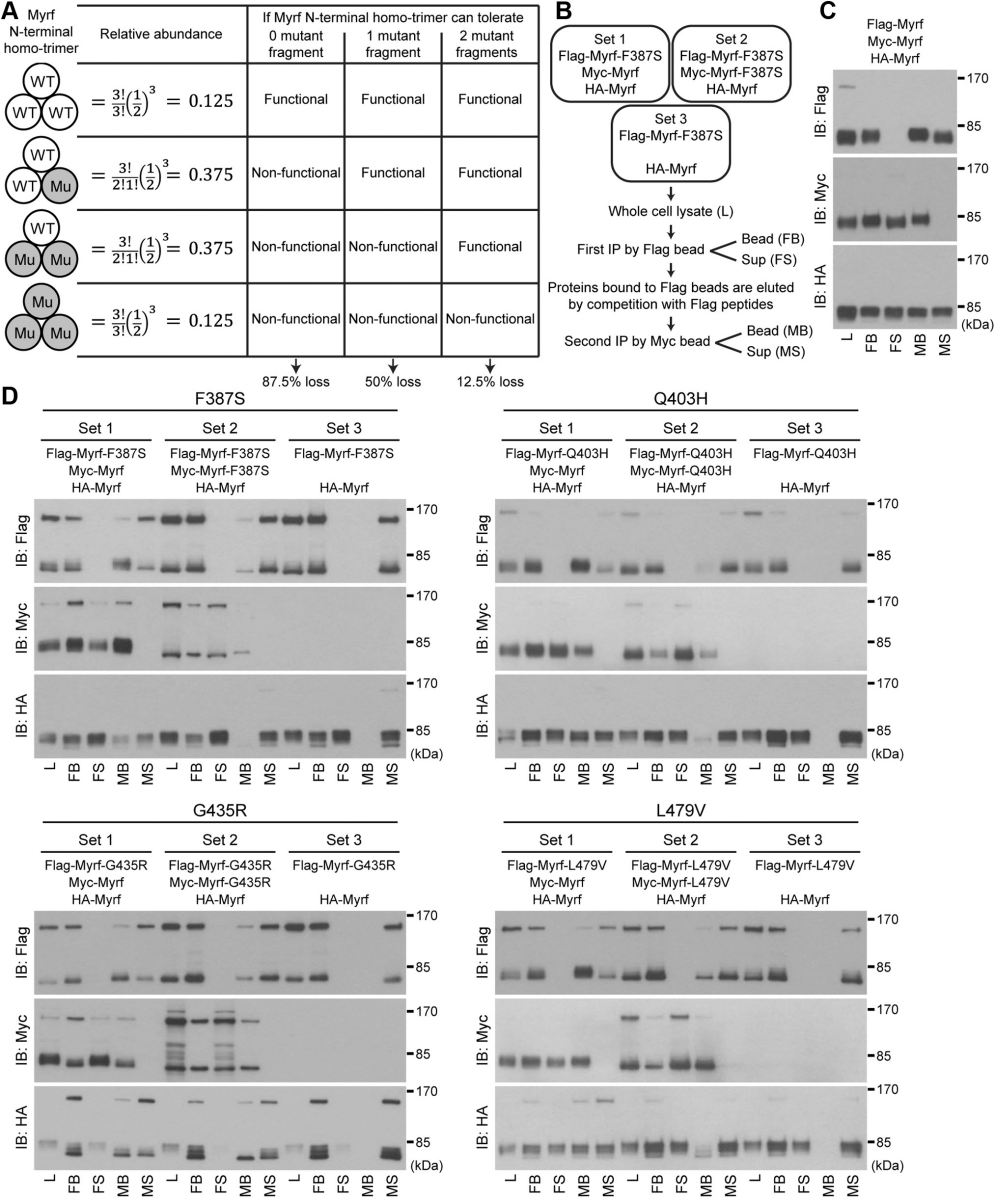
**Sequential immunoprecipitation of the four DBD mutants.***A*, relative portions of the four homo-trimeric species that are expected when wild-type and mutant alleles are expressed comparably. Impact of a mutant fragment on Myrf N-terminal homo-trimer is also indicated depending on how well the mutant is tolerated. *B*, sequential immunoprecipitation strategy. *C*, a control sequential immunoprecipitation experiment with wild-type Myrf demonstrating the effectiveness and specificity of sequential immunoprecipitation. *D*, sequential immunoprecipitation for the four DBD mutants. FB, proteins bound to Flag beads; FS, proteins not bound to Flag beads; IB, immunoblotting; L, cell lysate for the first immunoprecipitation with Flag beads; MB, proteins bound to Myc beads; MS, proteins not bound to Myc beads.

So far, we have elucidated the functional mechanisms of F387S, Q403H, G435R, and L479V. On this basis, an important issue to address now is how they cause birth defects in a heterozygous state. This is because they are all *de novo* mutations, which are present in patients as a heterozygote. Related to this is that Myrf N-terminal fragment strictly works as a homo-trimer, meaning that a loss-of-function mutation does not necessarily induce 50% loss. If a mutant *MYRF* allele is expressed at a comparable level to the wild-type one, 12.5% (=0.5^3^), 37.5% (=3 × 0.5^3^), 37.5% (=3 × 0.5^3^), and 12.5% (=0.5^3^) of MYRF N-terminal homo-trimers would contain 0, 1, 2, and 3 mutant fragments, respectively (Fig. 6*A*). If MYRF N-terminal homo-trimer does not tolerate even one mutant fragment, only 12.5% of MYRF N-terminal homo-trimers would be functional (Fig. 6*A*), leading to 87.5% loss. In contrast, if MYRF N-terminal homo-trimer tolerates as many as two mutant fragments, 87.5% of MYRF N-terminal homo-trimers would be functional (Fig. 6*A*). Thus, depending on how well a mutant fragment is tolerated by MYRF N-terminal homo-trimer, the impact of a mutant allele may be the same as, greater than, or less than 50% loss.

To determine how many mutant fragments are tolerated by Myrf N-terminal homo-trimer for its structural integrity, we performed sequential immunoprecipitation experiments for Myrf-F387S, Myrf-Q403H, Myrf-G435R, and Myrf-L479V, as in our previous study (26). For each mutant, we expressed three sets of plasmids in HEK293FT cells. Taking Myrf-F387S as an example, the three sets are as follows. Set 1 consisted of one mutant and two wild-type Myrf species (Flag-Myrf-F387S, Myc-Myrf, and HA-Myrf; Fig. 6*B*). Set 2 consisted of two mutant and one wild-type Myrf species (Flag-Myrf-F387S, Myc-Myrf-F387S, and HA-Myrf; Fig. 6*B*). Set 3 comprised a Flag-tagged mutant and an HA-tagged wild-type Myrf (Flag-Myrf-F387S and HA-Myrf; Fig. 6*B*), which was to check the specificity of immunoprecipitation with Myc beads. Whole-cell lysate (noted as “L” in Fig. 6*B*) was subject to immunoprecipitation with Flag beads, yielding the bead and sup fractions (“FB” and “FS” in Fig. 6*B*). Of note, the specificity of immunoprecipitation with Flag beads was demonstrated in Figure 3. Proteins bound to Flag beads were eluted by competition with Flag peptides. The eluate was subject to another round of immunoprecipitation with Myc beads, yielding the bead and sup fractions (“MB” and “MS” in Fig. 6*B*). The five fractions (“L,” “FB,” “FS,” “MB,” and “MS”) were blotted with Flag, Myc, and HA antibodies. By checking the presence of Myrf N-terminal fragment in each fraction, it is possible to determine how many mutant fragments are tolerated by Myrf N-terminal homo-trimer. Simply speaking, if HA-Myrf N-terminal fragment with the correct electrophoretic mobility pattern, which serves as a fingerprint for the structural integrity of Myrf N-terminal homo-trimer, appears in the MB fraction, it means that the Myrf species in the set are able to form intact homo-trimers.

To demonstrate the effectiveness and specificity of sequential immunoprecipitation, we performed a control experiment with wild-type Myrf (Fig. 6*C*). Consistent with the homo-trimerization of Myrf N-terminal fragment, we could detect HA-Myrf N-terminal fragment with the correct electrophoretic mobility pattern in the MB fraction (Fig. 6*C*). This was accompanied by Flag-Myrf and Myc-Myrf N-terminal fragments with the correct electrophoretic mobility pattern in the MB fraction, indicating a successful sequential immunoprecipitation. When the same sequential immunoprecipitation scheme was applied to the four DBD mutants, HA-Myrf N-terminal fragment with the correct electrophoretic mobility pattern was detected in the Set 1 MB fraction for F387S, Q403H, and L479V (Fig. 6*D*). These results indicate that one fragment of Myrf-F387S, Myrf-Q403H, or Myrf-L479V is tolerated by Myrf N-terminal homo-trimer without compromising structural integrity. In contrast, for G435R, HA-Myrf N-terminal fragment detected in the Set1 MB fraction showed only the fastest moving band, suggesting that its posttranslational modification induced by trimer formation was abolished and that the structure of HA-Myrf N-terminal fragment within the trimer was altered (Fig. 6*D*). To see if Myrf N-terminal homo-trimer can also tolerate two mutant fragments, we examined the Set 2 results (Fig. 6*D*). Either HA-Myrf N-terminal fragment was hardly detected in the MB fraction or it was present with an abnormal electrophoretic mobility. These observations suggest that Myrf N-terminal homo-trimer cannot tolerate two fragments of Myrf-F387S, Myrf-Q403H, Myrf-G435R, or Myrf-L479V. Of note, the Set 3 results validated the specificity of immunoprecipitation with Myc beads (Fig. 6*D*). Overall, we conclude that Myrf N-terminal homo-trimer can accommodate only one fragment of Myrf-F387S, Myrf-Q403H, or Myrf-L479V and that one Myrf-G435R fragment is sufficient to perturb the structural integrity of Myrf N-terminal homo-trimer.

### Functional impact of including Myrf mutants in Myrf N-terminal homo-trimer

**Figure 7:**
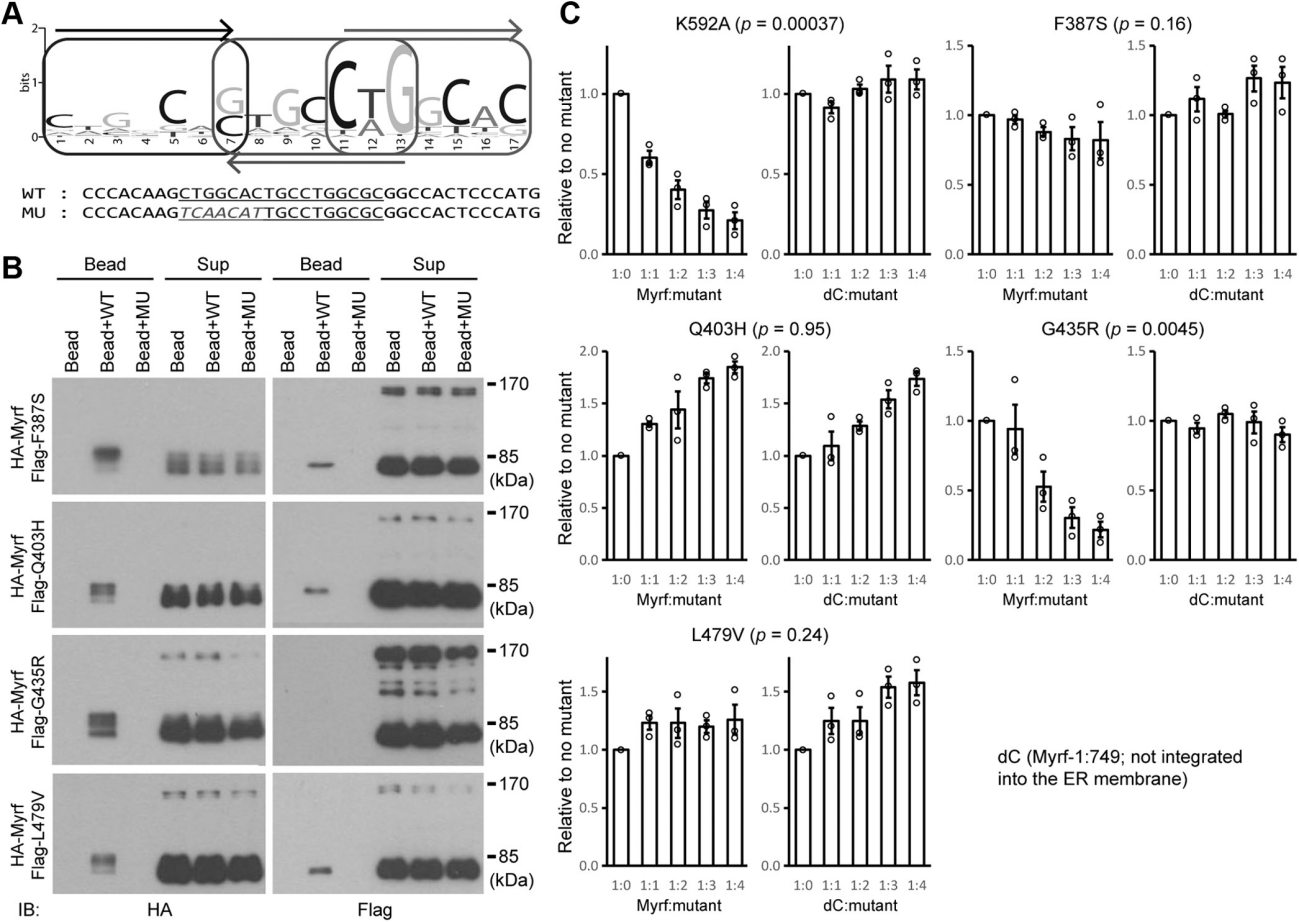
**DNA pull-down and luciferase assays for the four DBD mutants.***A*, the Myrf motif recognized by Myrf N-terminal homo-trimer. WT shows the DNA sequence used for the pull-down assay (rn4 chr10:71034513–71034549). Underlined is the Myrf motif incidence. MU shows a mutated sequence where five base pairs are mutated. *B*, DNA pull-down assay for the four DBD mutants. *C*, the effect of each mutant on the transcriptional activity of wild-type Myrf or dC. Increasing amounts of the mutant were coexpressed with wild-type Myrf or dC in Oli-neu cells, and their transcriptional activity was estimated by the reporter activity of Rffl. For each mutant, two-way ANOVA was performed, and the interaction *p* value indicated in the plot. Shown are data points and their mean and standard error. IB, immunoblotting.

The sequential immunoprecipitation experiment assessed the structural impact of including mutant fragments in Myrf N-terminal homo-trimer. We wanted to complement it with a functional investigation. To this end, we examined how the inclusion of a mutant in Myrf N-terminal homo-trimer affects its DNA binding. A 17 base pair-long DNA motif termed the Myrf motif mediates the sequence-specific DNA binding of Myrf N-terminal homo-trimer (22) (Fig. 7*A*). A key feature of the Myrf motif is that it consists of three degenerate copies of CTGGCAS (S being either G or C), with each consecutive pair arranged as a reverse complement. This architecture is in line with Myrf N-terminal fragment binding to DNA as a homo-trimer. The notable degeneracy observed in the Myrf motif suggests that for the DNA binding of Myrf N-terminal homo-trimer, not all three DBDs have to be engaged in DNA binding simultaneously. This raises the possibility that Myrf N-terminal homo-trimer that contains one mutant (F387S, Q403H, or L479V), which was above shown to be structurally intact, may be able to bind DNA in a sequence-specific manner.

To test this, we performed a DNA pull-down assay, as in our previous studies (22, 25). HA-Myrf and a Flag-tagged mutant (F387S, Q403H, G435R, or L479V) were coexpressed in HEK293FT cells, and cell lysates mixed with either bare beads or beads coated with duplex DNA oligos. The duplex oligo contained either a Myrf motif incidence found in the *Rffl* locus (“WT” in Fig. 7*A*) or a mutant version of it (“MU” in Fig. 7*A*). The mixture was separated into the sup and bead fractions by centrifuge, and both fractions were probed by HA and Flag antibodies to detect wild-type and mutant Myrf proteins, respectively. Immunoblotting with HA antibodies showed that wild-type Myrf N-terminal fragment avidly bound WT-coated beads in all samples (Fig. 7*B*). In contrast, it bound neither bare beads nor MU-coated beads, highlighting specificity toward the Myrf motif. To check whether Myrf N-terminal homo-trimer that bound WT-coated beads contained a mutant fragment, we probed the same samples with Flag antibodies. Mutant fragment was readily and specifically detected in the WT-coated bead for Myrf-F387S, Myrf-Q403H, and Myrf-L479V (Fig. 7*B*), indicating that Myrf N-terminal homo-trimer can tolerate one fragment of Myrf-F387S, Myrf-Q403H, or Myrf-L479V for sequence-specific DNA binding. In contrast, Myrf-G435R N-terminal fragment was hardly detected in the WT-coated bead, revealing that Myrf N-terminal homo-trimer that contains Myrf-G435R cannot bind the Myrf motif. This observation is consistent with the above sequential immunoprecipitation result that one fragment of Myrf-G435R is sufficient to disrupt the structural integrity of Myrf N-terminal homo-trimer. The sup fraction results showed that comparable amounts of proteins were used for the three binding reactions of each mutant Myrf, ruling out the trivial possibility that the specific detection of Myrf N-terminal fragment in the WT-coated bead is due to unequal protein amounts used for the binding reactions.

The sequential immunoprecipitation and DNA-binding assays indicate that Myrf-G435R, but not the other three mutants, works as a dominant negative. To confirm this, we performed a luciferase assay where we monitored the effect of increasing amounts of mutant Myrf on the transcriptional activity of wild-type Myrf. dC (a mutant Myrf truncated at the 750th residue) was used as a control for this assay. Since dC is truncated before the transmembrane domain, it is not integrated into the ER membrane (4), and its transcriptional activity is not affected by mutant Myrf species that are integrated into the ER membrane (22). Hence, dC allows us to estimate nonspecific effects associated with the expression of mutant Myrf. Before examining the four DBD mutants, we tested Myrf-K592A, a mutant that is known to act as a dominant negative (22). Two-way ANOVA (analysis of variance) revealed that Myrf-K592A significantly suppressed the transcriptional activity of Myrf, but not that of dC, as assessed by the reporter activity of Rffl (Fig. 7*C*, the interaction *p* value = 0.00037), confirming our experimental scheme. By the same analysis, we found that Myrf-G435R negatively affected the transcriptional activity of Myrf, but not that of dC (Fig. 7*C*, the interaction *p* value = 0.0045). The other three mutants had comparable effects on Myrf and dC. These results indicate that Myrf-G435R behaves as a dominant negative in terms of transcriptional activity, consistent with its phenotype in the sequential immunoprecipitation and DNA-binding assays. Taken together, we conclude that the G435R mutation acts as a dominant negative while the other three are simple loss-of-function variants.

## Discussion

Myrf is a pleiotropic membrane-bound transcription factor, playing a critical role in diverse organisms that range from human to slime mold (1, 2, 3, 4, 5, 6, 7). Myrf is a unique transcription factor for two reasons. First, to the best of our knowledge, it is the only membrane-bound transcription factor that undergoes auto-cleavage. Powered by the ICA domain, it forms a homo-trimer in the ER membrane and undergoes auto-cleavage to release its N-terminal fragment from the ER membrane as a homo-trimer. Second, Myrf N-terminal fragment strictly works as a homo-trimer. It enters the nucleus as a homo-trimer and binds to DNA as such for transcriptional regulation. Our previous study showed that homo-trimerization is essential for the transcriptional activity of Myrf N-terminal fragment because it imparts DNA-binding specificity and enables Myrf N-terminal fragment to tightly bind to the cognate DNA motif (the 17 base pair-long Myrf motif) (22). These two features (the ICA domain-catalyzed auto-cleavage and Myrf N-terminal fragment acting as an obligatory homo-trimer) form the backbone of Myrf biochemistry, which are conserved from human to slime mold. In this regard, it is no wonder that deleterious MYRF mutations implicated in birth defects hit these two core mechanisms. Of the six missense mutations uncovered so far, two (V679A and R695H) are mapped to the ICA domain. Our recent study has shown that they act by interfering with the auto-cleavage function of the ICA domain (26). The current study reports that the four mutations mapped to the DBD (F387S, Q403H, G435R, and L479V) act by disrupting the homo-trimerization of Myrf N-terminal fragment. The DBD crystal structure reveals that none of the four mutated residues is located in the interface between protomers (23), suggesting that impaired homo-trimerization is secondary to the disruption of the DBD structure itself. Since the start of this project, two more *MYRF* missense mutations have been associated with congenital anomalies (15, 31). Undoubtedly, future exome sequencing studies would uncover more mutations. Our studies provide unifying frameworks to understand the pathogenic mechanisms of *MYRF* mutations.

MYRF Q403R mutation has been linked to encephalopathy with reversible myelin vacuolization (19), a much milder condition compared with birth defects. It is interesting to note that two mutations affecting Q403 are associated with two disparate disorders. The original study reported that the transcriptional activity of MYRF-Q403R (equivalent to Myrf-Q403R) is about half that of wild-type MYRF (19). We were able to replicate this result, finding that Myrf-Q403R exhibits a significantly higher transcriptional activity than Myrf-Q403H and the other three DBD mutants studied here (unpublished observation). We also found that Myrf-Q403R N-terminal fragment is better able to maintain homo-trimerization (unpublished observation). These observations suggest that the phenotypic severity of MYRF DBD mutations is correlated with their impact on homo-trimerization and transcriptional activity.

According to the ExAC database, 11:61539012 TC (a variant *MYRF* allele that encodes a frameshift mutation, p.Ser264GlnfsTer74) is found in nominally healthy individuals at a high frequency (about 6.5 in 10,000) (28). This allele was also detected by another small-scale study (14). The transcript generated from 11:61539012 TC is likely to be subject to nonsense-mediated decay. Even if not, the protein that would be produced would be functionally null because it lacks all key functional domains. In sum, 11:61539012 TC is predicted to be a null allele. This allele was dismissed by Qi *et al.* as “homo-polymer artifacts” (13). As pointed out by them, the extra cytosine of 11:61539012 TC is found in a very long stretch of C’s, where sequencing errors often occur. Thus, the high frequency of 11:61539012 TC in the human population is likely due to sequencing errors. Other than 11:61539012 TC, there is no other high-frequency loss-of-function *MYRF* allele in nominally healthy individuals (28), indicating that *MYRF* is a highly constrained gene. It makes it likely that one copy of a loss-of-function *MYRF* allele is sufficient to cause congenital anomalies.

There are two ways for *MYRF* loss-of-function mutations to be pathogenic in a heterozygous state—haploinsufficiency and dominant negativity. Our sequential immunoprecipitation and DNA-binding and luciferase assays suggest that F387S, Q403H, and L479V act by haploinsufficiency, whereas dominant negativity comes into play for G435R. Structural analysis provides a plausible explanation about why G435R is the most detrimental. Arginine is much bulkier than glycine, and it is also positively charged. Two antiparallel β-sheets that pack against each other, which form a β-sandwich, are the core of the Myrf DBD structure (23, 24). The G435R mutation is likely to destroy one β-sheet irreversibly, blocking the proper folding of the Myrf DBD. The sequential immunoprecipitation result for Myrf-G435R suggests that while Myrf N-terminal homo-trimer that contains one Myrf-G435R fragment can somehow maintain homo-trimerization, its structural integrity is severely compromised. Consistently, Myrf N-terminal homo-trimer containing the G435R fragment could not bind to the Myrf motif, and Myrf-G435R negatively affected the transcriptional activity of Myrf in a manner expected of a dominant negative mutant. In addition, G435R also appears to impair the auto-cleavage reaction in the ER membrane. Taken together, one MYRF-G435R allele is expected to lead to 87.5% loss (Fig. 6*A*). In contrast, Myrf N-terminal homo-trimer appears to tolerate one fragment of Myrf-F387S, Myrf-Q403H, or Myrf-L479V for structural and functional integrity, limiting their impact to 50% loss.

## Experimental procedures

### Constructs

A *Myrf* cDNA that encodes the 1139-amino-acid-long mouse isoform was kindly provided by Dr Ben Emery (1). F387S, Q403H, G435R, and L479V (according to NM_001127392.2 (13)) are mapped to the same positions for this mouse Myrf cDNA, which was used for all the experiments reported in this study. The cDNA was cloned into pcDNA3 with an N-terminal Flag, Myc, or HA tag by using the In-Fusion cloning kit from Clontech. Point mutations were introduced by a PCR-based method. Rffl was generated by cloning a rat genomic fragment (rn4 chr10:71034166–71034749) into pGL3 promoter (Promega) (5, 22). The sequence information of all constructs was verified by Sanger sequencing.

### Cell culture

Oli-neu cells were kept in a proliferating condition by supplementing the Sato media (32) with PDGF (10 μg/ml), NT3 (1 μg/ml), CNTF (10 μg/ml), and NeuroCult SM1 Neuronal Supplement. They were maintained in a humidified 8% CO_2_ incubator at 37 °C. HEK293FT cells were cultured in Dulbecco’s modified Eagle’s medium supplemented with 10% fetal bovine serum and maintained in a humidified 5% CO_2_ incubator at 37 °C. Transient transfection was performed by using Lipofectamine 2000 as per the manufacturer’s instructions.

### Immunoblotting

Cells were rinsed once with PBS and lysed with 2X Laemmli Sample Buffer (Bio-Rad). Cell lysates were boiled at 95 °C for 5 min. Upon SDS-PAGE, proteins were transferred to PVDF and probed with horseradish peroxidase (HRP)-conjugated primary antibodies. The following dilutions were used for immunoblotting: mouse anti-Flag HRP-conjugated (Sigma #A8592, 1:5000), mouse anti-HA HRP-conjugated (Cell signaling #2999, 1:5000), mouse anti-c-Myc HRP-conjugated (Santa Cruz #sc-40, 1:2000), mouse anti-α-tubulin (Sigma #T9026, 1:50,000), and Gapdh (Sigma #G9545, 1:5000).

### Immunofluorescence

Cells were fixed with 4% formaldehyde and permeabilized with 0.1% Triton X-100. Upon blocking with 1% BSA, they were incubated with primary antibodies diluted in the blocking buffer at 4 °C overnight, followed by incubation with fluorochrome-conjugated secondary antibodies. Nuclei were stained with Hoechst 33342 (Invitrogen). Fluorescence was visualized with a Leica DMi8 microscope with an ORCA-Flash4.0 sCMOS camera. Reagents used for immunofluorescence are as follows: monoclonal anti-Flag M2 antibody (Sigma, 1:1000), goat anti-mouse calnexin antibody (Santa Cruz, 1:500), donkey anti-mouse antibody, Alexa Fluor 488 conjugate (Thermo Fisher, 1:5000), and donkey anti-goat antibody, Alexa Fluor 594 conjugate (ThermoFisher, 1:5000).

### Immunoprecipitation

Cells grown on 150 mm culture dishes were rinsed once with PBS, and 500 μl of 2X Cell Lysis Buffer (Cell Signaling) was added to them. Cell lysate was sonicated and spun down at 14,000 *g* for 10 min at 4 °C. Cleared cell lysate was mixed with antibody-coated beads (Sigma) and incubated for 2 h at 4 °C on a rotating plate. The mix was spun down at 7500 *g* for 30 s to separate it into supernatant and bead fractions. Sequential immunoprecipitation was performed as described previously (22).

### Luciferase assay

Luciferase assay was performed by using the Promega dual luciferase reporter assay kit as per the manufacturer’s instructions. Cells were cotransfected with a Myrf construct, Rffl, and pRL-TK (an internal control). The ratio between firefly and renilla luciferase activities was taken as the transcriptional activity of the Myrf construct.

### RT-qPCR

Total RNA was purified by using Trizol (Thermo Fisher Scientific #15596026), and cDNA synthesized by the SuperScript First-Strand kit (Invitrogen #11904–018). Quantitative PCR was performed on C1000 Touch thermal cycler with the CFX384 optical reaction module (Bio-rad). *Gapdh* was used for standard curves. Each PCR reaction contained 2 μl of cDNA, 5 μl of the iTaq Universal SYBR Green Supermix (Bio-rad #1725124), and 500 nM of forward and reverse primers. The primer sequences are as follows.

Myrf (forward): CCG CAT CAG CAG AAC AAG TGG G

Myrf (reverse): GCA TCG TCG CCC ACG GAA AAG

Plp1 (forward): GCC AGA ATG TAT GGT GTT CTC CCA TG

Plp1 (reverse): GGT GGA AGG TCA TTT GGA ACT CAG C

Gapdh (forward): GGT GAA GGT CGG TGT GAA CGG

Gapdh (reverse): CTG GAA CAT GTA GAC CAT GTA GTT GAG G

### DNA pull-down assay

HEK293FT cells were cotransfected with HA-Myrf and Flag-tagged mutant Myrf (F387S, Q403H, G435R, or L479V). Upon cell lysis, cell lysate was cleared by centrifugation at 15,000 *g* for 20 min at 4 °C. Biotinylated duplex oligonucleotides were conjugated to Dynabeads (Invitrogen) in buffer A (5 mM Tris pH 8.0, 0.5 mM EDTA, 1 M NaCl). Oligonucleotide-conjugated beads were washed twice with 500 μl of buffer A and three times with buffer C (20 mM Tris pH 8.0, 1 mM EDTA, 10% glycerol, 1 mM DTT, 50 mM NaCl). In total, 300 μg of cell lysate was incubated with oligonucleotide-conjugated beads in buffer C and sheared salmon sperm DNA (final concentration 0.2 mg/ml) for 20 min at room temperature with rotation. The mixture was spun down to separate the bead and sup fractions. The bead fraction was washed five times with 500 μl buffer C, and both fractions were analyzed by immunoblotting. The DNA sequences used are as follows. The Myrf motif is underlined, and the mutated portions are shaded in gray.

WT:TGACTACCCCACAAGCTGGCACTGCCTGGCGCGGCCA

MU:TGACTACCCCACAAGTCAACATTGCCTGGCGCGGCCA

## Data availability

All data that support the conclusion of the study are contained within the article.

## Supporting information

This article contains supporting information.

## Conflict of interest

The authors declare that they have no conflicts of interest with the contents of this article.
