# Supplementary material for: Functional mechanisms of MYRF DNA-binding domain mutations implicated in birth defects
Source: J Biol Chem. 2021 Mar 30;296:100612. doi: 10.1016/j.jbc.2021.100612 (PMC8094900; doi:10.1016/j.jbc.2021.100612)
Supplement: Figure S1 [file mmc1.docx]

**Functional mechanisms of MYRF DNA-binding domain mutations implicated in birth defects**

Chuandong Fan^1#^, Hongjoo An^1#^, Mohamed Sharif^1^, Dongkyeong Kim^1^, and Yungki Park^1,^*

^1^Hunter James Kelly Research Institute, Department of Biochemistry, Jacobs School of Medicine and Biomedical Sciences, State University of New York at Buffalo, Buffalo, NY 14203, USA

*To whom correspondence should be addressed.

Tel: 1-716-881-7579; Fax: 1-716-849-6651; Email: [yungkipa@buffalo.edu](mailto:yungkipa@buffalo.edu)

^#^Equal contribution (co-first authors)

**Running title:** Disease mechanisms of MYRF DBD mutations

**Keywords:** MYRF, membrane-bound transcription factor, transcription factor, mutation, birth defects, disease mechanism


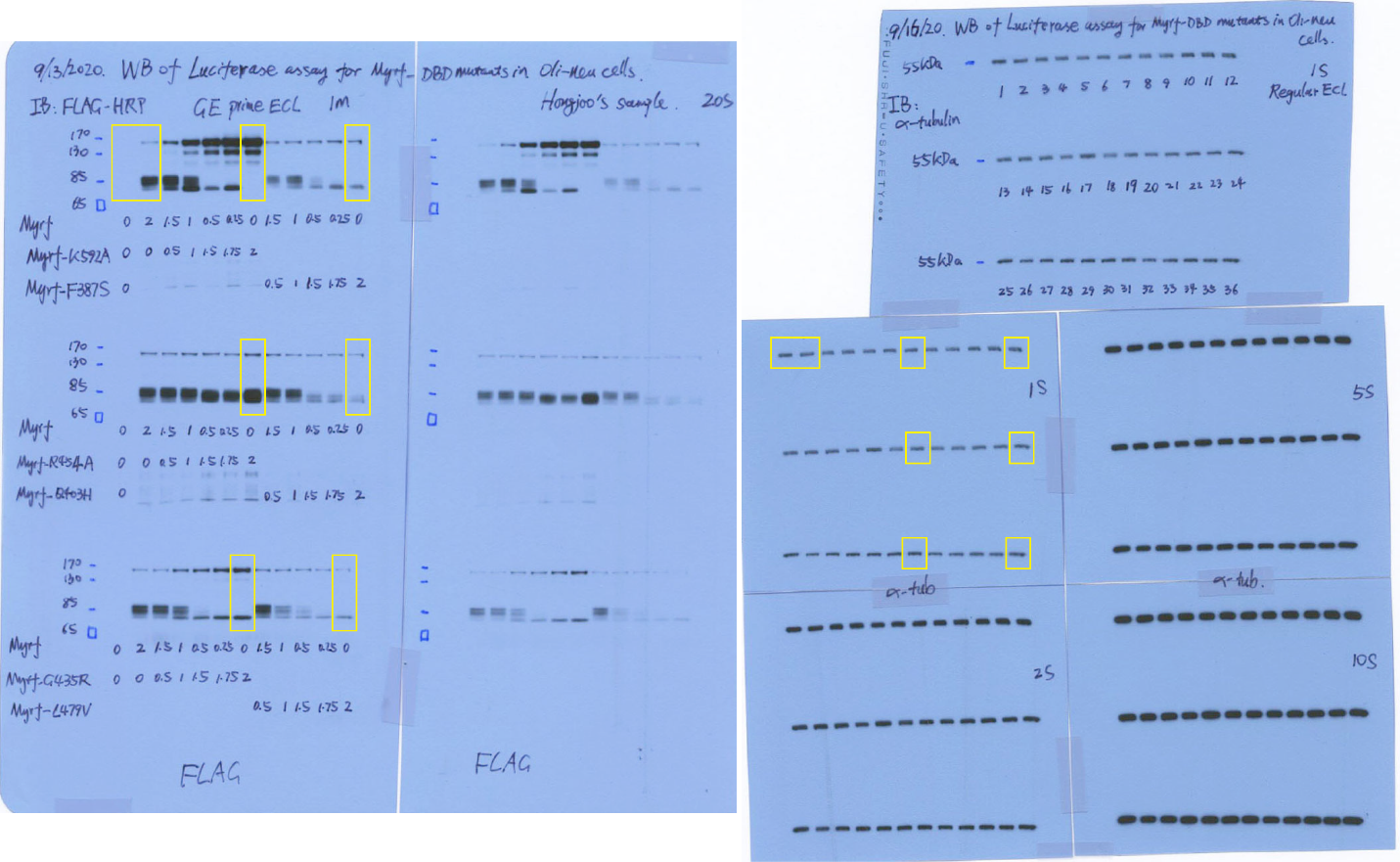


**Figure S1**

The raw Western blot results for **Figure 4B**. Cropped portions are marked by yellow boxes.
